# Level of knowledge, attitudes and beliefs towards patients with chronic low back pain among final year School of Therapeutic Sciences students at the University of the Witwatersrand – A cross-sectional study

**DOI:** 10.4102/sajp.v75i1.683

**Published:** 2019-08-14

**Authors:** Grace Mukoka, Benita Olivier, Sadiya Ravat

**Affiliations:** 1 Department of Physiotherapy, School of Therapeutic Sciences, Faculty of Health Sciences, University of the Witwatersrand, Johannesburg,South Africa; 2 Department of Physiotherapy, College of Medicine, University of Malawi, Blantyre, Malawi

**Keywords:** knowledge of pain, attitude, beliefs, patients with chronic low back pain, undergraduate students

## Abstract

**Background:**

Knowledge of neurophysiology of pain influences healthcare providers’ attitudes and beliefs about patients with chronic low back pain which affect management choices.

**Objectives:**

The aim of this study was to determine the level of knowledge of pain and attitudes and beliefs towards patients with chronic low back pain among final year undergraduate students from the School of Therapeutic Sciences at the University of the Witwatersrand.

**Methods:**

This cross-sectional study included two questionnaires – Health Care Providers’ Pain and Impairment Relationship Scale (HC-PAIRS) for measuring attitudes and beliefs about pain and the Neurophysiology of Pain Questionnaire (NPQ) for knowledge of pain. These were distributed to 224 students. An analysis of variance and a two-sided *t* tests compared data with *p* ≤ 0.05.

**Results:**

The study had a 65% response rate (*n* = 145), of which the majority were female students (*n* = 115, 79%). Overall, the mean correct NPQ score was 6.01 (± 1.98), with a significant difference among the programmes (*p* = 0.005). Mean NPQ scores for each programme were as follows: physiotherapy 6.97 (1.77), biokinetics 6.31 (2.43), exercise science 6.25 (2.5), pharmacy and pharmacology 5.69 (1.39), nursing 5.32 (1.39) and occupation therapy 5.21 (2.09). The mean correct scores for HC-PAIRS were 63.1 (8.9), with significantly higher scores in females than males (*p* = 0.04). Knowledge scores had a low inverse relationship with scores for attitudes and beliefs towards patients with chronic low back pain (*r* = -0.304; *p* = 0.0002).

**Conclusion:**

There is a deficit in knowledge of pain among final year students in the School of Therapeutic Sciences, with a low correlation with attitudes and beliefs towards patients with chronic low back pain. Therefore, improving the knowledge of pain might result in a change in these attitudes and beliefs.

**Clinical implications:**

The results have shown an association between knowledge of pain and attitudes and beliefs towards patients with chronic low back pain. Therefore, knowledge is one of the factors that could contribute in changing the attitudes.

## Introduction

Low back pain is one of the leading causes of disability worldwide (Hoy et al. 2010). Most cases of acute low back pain progress to chronicity when recovery takes more than 3 months. This is associated with psychosocial factors (anxiety, stress, patients’ recovery expectations, somatisation, depression and fear avoidance behaviour), brain structural changes (an increase in cortical thickness, expansion and medially shifting of homunculus) and neurochemical changes (a decrease in N-acetyl-aspartate, glutamate, glucose level, choline and myo-inositol), which lead to changes in the central mechanism of pain (Stubbs et al. 2016; Zhao et al. 2017).

The management approach to chronic low back pain has evolved from a biomedical to a biopsychosocial approach to consider the psychosocial factors of patients with chronic low back pain (O’Sullivan 2012; Wijma et al. 2016). A multidisciplinary team and biopsychosocial approach are currently recommended in managing patients with chronic low back pain to gain optimal results (Luk et al. 2010). However, there is poor uptake of the biopsychosocial approach by healthcare providers because of their negative attitudes towards patients with chronic low back pain, poor knowledge of the neurophysiology of pain and their focus on biomedical factors, patient perception and cost (Dwyer et al. 2017). Knowledge of pain and attitudes and beliefs about chronic low back pain among healthcare providers play a vital role in the choice of approach for the management of patients with chronic low back pain (Dwyer et al. 2017; Nijs et al. 2013). Education of the neurophysiology of pain has been effective in shifting the attitudes and beliefs of healthcare providers more positively, hence ensuring success in implementation of the biopsychosocial approach (Domenech et al. 2011).

Most undergraduate students in the School of Therapeutic Sciences at the University of the Witwatersrand are exposed to the clinical environment, where they are expected to manage patients with chronic low back pain. Their knowledge of the neurophysiology of pain and their attitudes and beliefs about patients with pain are equally important in determining the appropriate approach in the management of patients with chronic low back pain.

Healthcare providers’ level of knowledge of the neurophysiology of pain is reported to influence their attitudes and beliefs towards patients with chronic low back pain, which then affects their assessment of and treatment approach to patients. This level of knowledge is found to differ across different professions because of a number of factors, that is, different curricula and number of years of experience. In South Africa, the level of knowledge of pain assessed among practising therapists is reported to be poor (Clenzos, Naidoo & Parker 2013). However, no research regarding this has been done among undergraduate students in South Africa. Therefore, the objectives of this study were to determine the level of knowledge of pain, the attitudes and beliefs about patients with chronic low back pain and the relationship between knowledge of pain and attitudes and beliefs of students towards patients with chronic low back pain.

## Method

This cross-sectional study was conducted among final year students from the School of Therapeutic Sciences at the University of the Witwatersrand. There were a total of 224 final year students who were enrolled and registered for the 2017 academic year. These included 54 physiotherapy students, 44 occupational therapy students, 26 nursing students, 61 pharmacy and pharmacology students, four exercise science and sports medicine students and 35 biokinetics students.

### Measuring tools

The revised version of the Neurophysiology of Pain Questionnaire (NPQ) was used to measure the level of knowledge of the neurophysiology of pain (Catley, O’Connell & Moseley 2013). It has a test–retest reliability of 0.84 (Catley et al. 2013). It has 12 items, with each item to be indicated as either true (T), or false (F) or undecided (U). Correct responses were awarded 1 point, and incorrect or undecided responses were awarded 0 points. Therefore, the score ranges from 0 to 12. The higher the NPQ scores, the better the understanding of the neurophysiology of pain.

The Health Care Providers’ Pain and Impairment Relationship Scale (HC-PAIRS) was used to collect data on attitudes and beliefs towards patients with chronic low back pain (Rainville, Bagnall & Phalen 1995). Its internal consistency ranges from 0.78 to 0.83 and criterion validity has been tested. It contains 15 items, each item being rated on a 7-point Likert scale: 1 for completely disagree and 7 for completely agree. The scores range from 15 to 105. The higher the scores, the stronger the belief that chronic pain justifies impairment and disability (Rainville et al. 1995).

Participants were asked to provide their demographic details, which included age, gender, programme of study, current and previous history of pain.

### Data collection procedure

The first author visited the participants’ classrooms at a time convenient to them (ensuring not to interfere with their studies), to explain the purpose of the study and to invite them to participate. Three self-administered questionnaires (demographic questionnaire, NPQ and HC-PAIRS) were distributed to the students to complete in hard copy. The participants were invited to drop the completed questionnaires in a sealed box to ensure anonymity.

### Data analysis

Data were analysed using STATA IC version 14.1. Age, NPQ scores and HC-PAIRS scores were assessed for normal distribution using the Shapiro–Wilk test. Subsequently, a comparison of NPQ score and HC-PAIRS score between two groups (gender: male and female, age: ≤ 22 and > 22, with and without current history of low back pain, and with or without past history of low back pain) was done using a two-sided *t* test. An analysis of variance (ANOVA) was used to compare mean scores across the programmes of study. Tukey *post hoc* test was used for ANOVA where a significant difference was observed. A pairwise correlation test was used to measure the correlation between NPQ mean scores and HC-PAIRS mean scores.

Categorical data were summarised as proportions and percentages, while means (standard deviation [s.d.]) were used to summarise continuous data. The significance level was set at a two-sided alpha level of 0.05.

### Ethical considerations

This study obtained ethical clearance (ref. no. M170615) from the University of the Witwatersrand’s Human Research Ethics Committee before commencement. It was conducted in accordance with the principles of the 2013 Declaration of Helsinki. The study received permission from the University Deputy Registrar, the Head of the School of Therapeutic Sciences and heads of the involved units.

Information sheets were distributed to the study population, which informed them about the purpose of the study, their role and their rights in the study. The demographic details were collected and did not include any identifiable information. Participants’ responses were anonymous and were only used for this study’s purpose.

## Results

### Demographics characteristics

There was an overall response rate of 65% (*n* = 145), with exercise science having the lowest representation (*n* = 4, 2.8%) (Table 1). The mean age was 22.6 (± 1.39) years and there were more females (*n* = 115, 79%) than males (*n* = 30, 20.69%). Few students had a current history of low back pain (*n* = 41, 28%).

**TABLE 1:** Demographic characteristics and measurement tools (*n* = 145).

Demographic characteristics	Frequency	%	NPQ scores	s.d.	*p* (for NPQ)	HC-PAIRS scores	s.d.	*p* (for HC-PAIRS)
**Overall**	145	65	6.01	1.98		63.1	8.9	
**Age**					0.290			0.41
≤ 22 years	79	54.48	5.85	2.15		62.58	8.22	
> 22 years	66	45.52	6.19	1.74		63.80	9.74	
**Gender**					0.050*			0.04*
Male	30	20.69	6.63	1.83		60.1	8.74	
Female	115	79.31	5.84	1.99		63.93	8.85	
**Programme of study**					0.005*			0.14
Biokinetics	32	22.07	6.31	2.43		60.41	6.63	
Exercise science	4	2.76	6.25	2.5		59.25	13.23	
Nursing	22	15.17	5.32	1.39		63.91	10.78	
Occupational therapy	24	16.55	5.21	2.09		65.63	10.20	
Pharmacy and pharmacology	29	20.00	5.69	1.39		65.41	8.64	
Physiotherapy	34	23.45	6.97	1.77		61.97	7.83	
**Current history of low back pain:**					0.800			0.16
Yes	41	28.28	5.07	2.22		61.46	9.36	
No	104	71.72	5.98	1.88		63.80	8.72	
**Past history of low back pain**					0.600			0.48
Yes	104	71.72	5.95	2.05		63.47	8.65	
No	41	28.28	6.15	1.78		62.29	9.67	

* , *P* ≤ 0.05.

s.d., standard deviation; HC-PAIRS, Health Care Providers’ Pain and Impairment Relationship; NPQ, Neurophysiology of Pain Questionnaire.

### The revised version of Neurophysiology of Pain Questionnaire

The overall NPQ mean score was 50%, indicated by a score of 6.01 out of 12 (Table 1). Neurophysiology of Pain Questionnaire mean scores were significantly different across programme of study (ANOVA test, *p* = 0.005). Physiotherapy had a significantly higher NPQ mean score than nursing (Tukey test, *p* = 0.02) and occupational therapy (Tukey test, *p* = 0.01). Female students had significantly lower mean scores on the NPQ questionnaire 5.84 (± 1.99) than male students 6.63 (± 1.83) (*t* test, *p* = 0.05). There was no significant difference in NPQ mean scores by age, current or past history of low back pain (Table 1).

### Health Care Providers’ Pain and Impairment Relationship Scale

The overall HC-PAIRS, as a measure of attitudes and beliefs, mean score was 63.1 (± 8.9) out of 105. There was statistical difference in HC-PAIRS mean score by gender (*t* test, *p* = 0.04), with females having a higher score (63.93 ± 8.85) than males (60.63 ± 1.83) (Table 1). There was no significant difference in HC-PAIRS mean scores by age, programme of study and history of low back pain.

**FIGURE 1:**
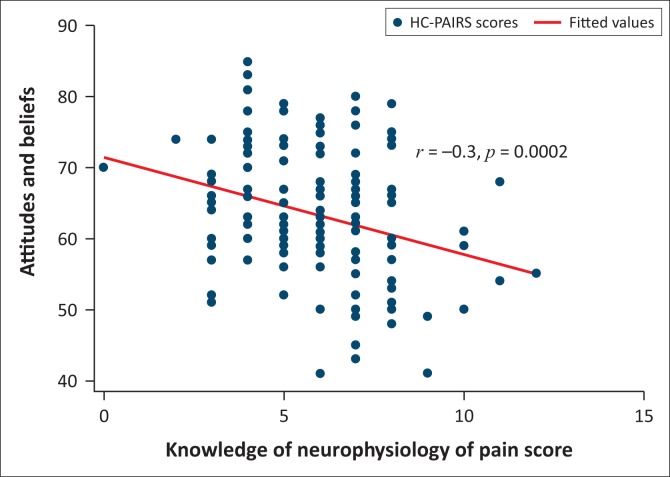
Neurophysiology of Pain and Health Care Providers’ Pain and Impairment Relationship Scale: Pairwise correlation scatter plot.

Figure 1 shows a relationship between knowledge of the neurophysiology of pain and attitudes and beliefs towards patients with chronic low back pain. There was a decrease of NPQ scores with an increase in HC-PAIRS scores.

## Discussion

This study set out to determine the level of knowledge of pain, the attitudes and beliefs about patients with chronic low back pain and the relationship between knowledge of pain and attitude and beliefs of students towards patients with chronic low back pain. Overall, the level of knowledge of pain among these students can be considered as average, with physiotherapy students displaying a higher NPQ score when compared to nursing and occupational therapy students. Male students had better knowledge of pain, while female students had more positive attitudes and beliefs towards patients with chronic low back pain. Knowledge of pain had an inverse low correlation with attitudes and beliefs about patients with chronic low back pain.

The overall NPQ mean percentage score for this study was 50%, which is considered average when compared to Adillón, Lozano and Salvat’s (2015) study. They found an NPQ mean percentage of 42.14% among first year health science students and 58.13% among final year health science students. Undergraduate health science students have a better understanding of the neurophysiology of pain in their final year than in their first year of study (Adillón et al. 2015).

Our students believed that chronic pain justifies impairment and disability and are likely to give advice which favours a biomedical approach, that is, bed rest and avoiding painful movements. Evidence shows that there is a significant change in attitudes along the course of study where final year health science students have positive attitudes about patients with chronic low back pain compared to first year students (Kennedy, Healy & O’Sullivan 2014). This is because final year students will have learnt about the neurophysiology of pain. However, our study cannot deduce whether the students’ attitudes and beliefs have changed along their course of study because students from the junior years were not included and hence could not be compared. According to Clenzos et al. (2013), therapists are reported to have inadequate knowledge of the neurophysiology of pain. This implies that even after learning about pain during undergraduate programmes, healthcare providers still have a deficit in their knowledge of the neurophysiology of pain. Therefore, reviewing the pain curricula content of health science programmes would be of value.

Programme of study was associated with the knowledge of pain among these students. Physiotherapy, biokinetics and exercise science students had higher scores for knowledge of pain than pharmacy and pharmacology, nursing and occupational therapy students. This indicates that the knowledge of pain is different across programmes of study, and can be attributed to differences in pain curriculum content. Further studies regarding the pain curricula would help to elaborate how much they differ and how they contribute to students’ attitudes towards patients with chronic low back pain. This would be important for physiotherapy and occupational therapy professions who often engage with patients experiencing chronic pain. In a systematic review by Ung et al. (2016), all health science students were found to have poor knowledge of pain; however, physiotherapy students had significantly better knowledge of the neurophysiology of pain than nursing and medical students.

An intervention study done among health science students showed that exposure to biopsychosocial education improves their knowledge of pain and attitudes compared to biomedical education (Domenech et al. 2011). Hence, the difference in the knowledge of pain among our students could be because of whether their curriculum employs a biopsychosocial or biomedical approach. The level of understanding that can be expected from undergraduate students has not been explored. Because these students have had minimal exposure to patients with chronic pain, it is possible that the level of knowledge will improve with increasing clinical exposure to patients with low back pain. No similar studies have been done on biokinetics and exercise science students. Here their levels of knowledge of pain were similar to those of physiotherapy students. However, caution should be applied when interpreting these results given the biokinetics and exercise science students’ representation was small.

Gender was associated with knowledge of the neurophysiology of pain, where male students had significantly higher knowledge of the neurophysiology of pain than females. The proportion of male students in this study was small (21%). In a study by Adillón et al. (2015), the number of male students (33%) was also disproportional to that of female students (67%). Their study considered the knowledge of the neurophysiology of pain among first and final year students who were pursuing medicine, physiotherapy and nutrition degrees at Rovira i Virgili University in Spain. They also found a significantly higher NPQ scores in males than females, indicating that male students have better knowledge of pain than females. It is unclear why male students have a better knowledge of pain. Future studies should explore the relationship between knowledge of pain and gender.

While knowledge of pain differed for the programmes of study, no statistical difference in attitude and beliefs across programme of study was found. This is contrary to the evidence that report different attitudes and beliefs among students in various health science programmes (Burnett et al. 2009). This could be because our study recruited students in their final year of the programme and all of them had been exposed to low back pain education.

Interrogating the curricula of each programme of study may provide valuable insights. However, this was outside the scope of our study. Briggs et al. (2013) also found a significant difference in beliefs of back pain between various programmes of study using HC-PAIRS to assess the beliefs of students and their alignment with published studies. In addition, they assessed the spinal curricular content across programmes where they found that physiotherapists and chiropractors had a bigger volume and emphasis on spinal pain in their curricula which translated to their more helpful beliefs about back pain compared to pharmacy, medicine and occupational therapy (Briggs et al. 2013).

Female students had higher mean score on the HC-PAIRS questionnaire than males. This shows that females are more likely to believe that chronic pain justifies impairment and disability than males. Therefore, when dealing with chronic pain, females will favour a biopsychosocial approach. There is some controversy in the literature on whether there is an association between gender and attitudes. Some studies report that gender has an association with attitudes and beliefs (Kennedy et al. 2014; Magalhães et al. 2012), while another study reports no association (Ryan et al. 2010).

Kennedy et al. (2014) in their study on Irish undergraduate students had a low response rate, with 78% female representation and 22% males using the Back Beliefs questionnaire and Fear Avoidance Beliefs questionnaire physical sub-section as outcome measures. The differences in outcome measures used make direct comparisons to our study challenging. Magalhães et al. (2012) included qualified physiotherapists in a similar study with the Pain Attitudes and Beliefs for Physiotherapists and HC-PAIRS as outcome measures, but the extent of exposure of the sample to chronic pain will have had an effect. Morris et al. (2012) found no statistical difference in HC-PAIRS scores between female and male health science and non-health science students.

There was no significant difference in HC-PAIRS mean score by history of low back pain among our participants. This finding comes as a surprise because it is expected that someone with a history of low back pain may have better empathy and therefore more positive attitudes and beliefs towards low back pain. Contrary to this finding, a history of low back pain was associated with better attitudes and beliefs towards low back pain among Brazilian and Ghanaian physiotherapy students (Ferreira et al. 2004).

We found a low negative correlation between knowledge of pain and attitudes and beliefs towards patients with chronic low back pain. This indicates that the higher the students’ knowledge regarding pain, the better their attitudes towards patients with chronic low back pain and the higher their likelihood may be of using a biopsychosocial model over a biomedical model.

An increase in the knowledge of pain improves the attitudes and beliefs towards patients with chronic low back pain among physiotherapy students (Colleary et al. 2017). When physiotherapy students are assigned to biopsychosocial training as opposed to biomedical training, their attitudes and beliefs towards patients improve (Domenech et al. 2011). Education about the neurophysiology of pain seems to be important in changing the attitudes and beliefs of students towards patients with chronic low back pain.

## Limitations

This study used the HC-PAIRS questionnaire to assess the level of attitudes and beliefs towards patients with chronic low back pain. Its validity and reliability have been confirmed among health professionals, but have not been established among students. This questionnaire does not have a cut point score for those who believe that pain justifies impairment. Therefore, in this study, the interpretations of attitudes were made based on similar studies. In addition, being a cross-sectional study, the interpretations of results were limited in terms of association between variables.

## Conclusion

This study shows that final year students have a deficit in knowledge of the neurophysiology of pain and believe that chronic pain justifies impairment. Knowledge of the neurophysiology of pain was significantly different across programmes and gender. The participants in this study presented with negative attitudes and beliefs, with females having significantly more negative attitudes and beliefs towards patients with chronic low back pain than males.

The findings from this study have important practical implications. The curricula of the different therapeutic sciences programmes should be scrutinised with regard to the chronic pain content, including management thereof. An interdisciplinary neurophysiology of pain education for all health science programmes may be of benefit. This may ensure that the knowledge of pain will be improved and may contribute to more positive attitudes and beliefs towards chronic low back pain. Overall, the implication of such programmes will be that the biopsychosocial model may be favoured when managing patients with chronic low back pain.
